# Appendicitis and Presence of a Ventriculoperitoneal (VP) Shunt

**DOI:** 10.7759/cureus.23491

**Published:** 2022-03-25

**Authors:** David R Hallan, Hanel Eberly, Elias Rizk

**Affiliations:** 1 Neurosurgery, Penn State Health Milton S. Hershey Medical Center, Hershey, USA

**Keywords:** mortality rate, shunt revision, outcomes, ventriculoperitoneal shunt, shunt, appendicitis, hydrocephalus, neurosurgery

## Abstract

Introduction: Appendicitis can cause ventriculoperitoneal (VP) shunt infection. However, little data is available on the incidence of shunt infections and shunt revisions with appendicitis. Therefore, we sought to determine the rates of shunt infection and revision in patients with VP shunt and appendicitis using large database data and review the literature.

Methods: We used a de-identified database network (TriNetX) to retrospectively query via ICD-10 and current procedural terminology codes to evaluate all patients with the presence of a VP shunt and appendicitis. Primary outcomes included shunt infection and shunt revision at 90 days, with secondary outcomes of sepsis and seizure.

Results: 396 patients with VP shunt and subsequent appendicitis were identified. The average age was 27.02+-20.94 years. Shunt infection was identified in 43 (10.859%) patients within 90 days of appendicitis, and shunt externalization or revision was performed in 66 (16.667%) patients. Sepsis was identified in 49 (12.374%) patients and seizures occurred in 56 (14.141%) patients. The literature review revealed eight relevant articles, with 49 total patients. Ten (20.408%) patients had shunts externalized, four of which occurred after shunt infection was identified. Shunt infection occurred in a total of 11 (22.449%) patients. Two (4.082%) patients died, one of which had their shunt externalized pre-emptively, and the other after ventriculitis was identified. Shunt revisions were performed in 16 (32.653%)

Conclusion: Our results demonstrate that shunt externalization should be strongly considered in patients with appendicitis, given high shunt infection rates.

## Introduction

Infections are thought to be one of the most common causes of shunt failure [1]. Appendicitis is a particularly common abdominal infection, and acute appendicitis is the most common condition requiring emergency operation [2,3]. Rupture of an appendix can lead to spread of infection throughout the abdomen. Due to the shunt’s location in the abdomen, an abdominal infection can have especially grave consequences such as CNS infection, diminished shunt function or shunt failure, and increased intracranial pressure [4]. Yet there is little data connecting the incidence of appendicitis and ventriculoperitoneal (VP) shunt infection, or necessity of VP shunt revision or externalization. The purpose of this study is to leverage large database data to determine how common shunt infection and shunt revision is following appendicitis in order to guide practice.

## Materials and methods

This was a retrospective comparative case-control study. We used a de-identified database network (TriNetX) to retrospectively query via ICD-10 and current procedural terminology codes to evaluate all patients with the presence of a VP shunt and appendicitis. Data came from 56 health care organizations (HCOs) spanning six countries and over 78 million patients. Data includes demographics, diagnoses, medications, laboratory values, genomics, and procedures. The identity of the HCOs and patients are not disclosed to comply with ethical guidelines against data re-identification. Because of the database's federated nature, an institutional review board (IRB) waiver has been granted. The data is updated daily. Our use of this database and its validity was informed by previous literature, and exact details of the network have been previously described [5-8].

Medical information included age at index date, as well as sex, race, and comorbidities of hypertension, acute kidney injury, diabetes, ischemic heart disease, heart failure, atrial fibrillation, disorders of lipoprotein metabolism and other lipidemias, obesity, history of nicotine dependence, chronic respiratory disease, cirrhosis, alcohol abuse or dependence, and peripheral vascular disease, recorded up to the date of the index date. Our primary outcomes included shunt infection and shunt revision at 90 days, with secondary outcomes of sepsis and seizure. Chi-square analysis was performed on categorical variables.

## Results

396 patients were identified. Average age was 27.02+-20.94 years. 289 patients (72.613%) were white, 56 (14.07%) black or African American, and <10 patients (<2.513%) were Asian. 214 (53.769%) of patients were male. Data was unable to be collected as to whether the appendicitis was classified as ruptured or unruptured.

Baseline demographics and characteristics are shown in Table 1.

**Table 1:** Baseline demographics and characteristics

Code	Diagnosis	Cohort 1, n (%)
AI	Age at Index	27.02 (100)
2106-3	White	289 (72.613)
M	Male	214 (53.769)
F	Female	184 (46.231)
2054-5	Black or African American	56 (14.07)
2131-1	Unknown Race	45 (11.307)
2028-9	Asian	10 (2.513)
I10-I16	Hypertensive diseases	117 (29.397)
R53	Malaise and fatigue	117 (29.397)
J40-J47	Chronic lower respiratory diseases	98 (24.623)
R63	Symptoms and signs concerning food and fluid intake	98 (24.623)
E78	Disorders of lipoprotein metabolism and other lipidemias	68 (17.085)
R13	Aphagia and dysphagia	60 (15.075)
R40	Somnolence, stupor and coma	50 (12.563)
N17-N19	Acute kidney failure and chronic kidney disease	46 (11.558)
E08-E13	Diabetes mellitus	41 (10.302)
F17	Nicotine dependence	37 (9.296)
I20-I25	Ischemic heart diseases	34 (8.543)
Z87.891	Personal history of nicotine dependence	32 (8.04)
I50	Heart failure	26 (6.533)
I48	Atrial fibrillation and flutter	12 (3.015)
K74	Fibrosis and cirrhosis of liver	<10 (<2.513)
F10.1	Alcohol abuse	<10 (<2.513)
F10.2	Alcohol dependence	<10 (<2.513)
I73	Other peripheral vascular diseases	<10 (<2.513)

Table 2 shows outcomes. Shunt infection was identified in 43 (10.859%) of patients, with sepsis identified in 49 (12.374%) patients. Shunt revisions occurred in 66 (12.374%) of patients. Seizures occurred in 56 (14.141%) of patients.

**Table 2:** Outcomes

Outcome	Cohort 1, n (%)
Shunt infection	43 (10.859)
Shunt revision	66 (16.667)
Sepsis	49 (12.374)
Seizures	56 (14.141)

**Figure 1:**
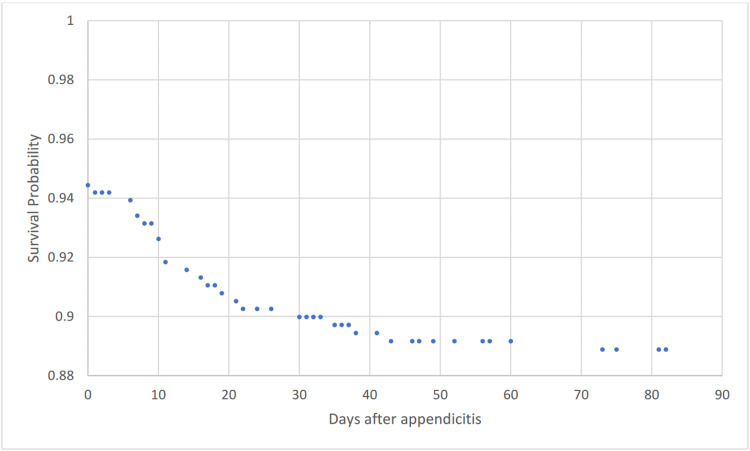
Kaplan-Meier survival analysis for outcome shunt infection

Figure 1 shows a Kaplan-Meier survival curve for outcome shunt infection to 90 days.

**Figure 2:**
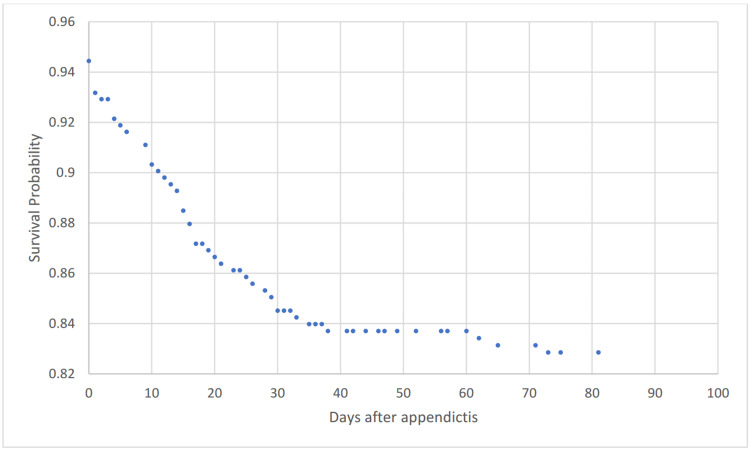
Kaplan-Meier survival analysis for outcome shunt revision

Figure 2 shows a Kaplan-Meier survival curve for outcome shunt revision to 90 days.

## Discussion

Few studies have come to a consensus on the necessity of VP shunt revision or externalization for acute appendicitis. The incidence of VP shunt infections from appendicitis is unknown. A literature review revealed eight relevant articles, with 49 total patients. Ten (20.408%) patients had shunts externalized, four of which occurred after shunt infection was identified. Shunt infection occurred in a total of 11 (22.449%) patients. Two (4.082%) patients died, one of which had their shunt externalized pre-emptively, and the other after ventriculitis was identified. Shunt revisions were performed in 16 (32.653%) patients [4,9-15].

Ein and colleagues studied eight children with appendicitis. Three patients had perforated appendicitis (37.5%) and five had non-perforated appendicitis (62.5%). All eight patients underwent an appendectomy immediately following diagnosis. The VP shunt was externalized for the three perforated cases. The shunts were not externalized in the non-perforated cases. Follow-up ranged from one to 30 years. Four children (three with acute appendicitis and one with ruptured appendix) had one-to-three shunt revisions from three months to four years after appendectomy. Ein and colleagues concluded that the VP shunt must be temporarily exteriorized in the case of appendiceal rupture [10].

Häussler and colleagues described a series of 21 children with appendicitis. Seven patients had a non-perforated appendix, five patients with ulcerous appendicitis, five patients with chronic appendicitis, and four patients had a perforated appendix. Nine (42.9%) patients developed a cerebrospinal fluid (CSF) infection, including 100% of the patients with perforated appendicitis. None of the patients were initially externalized. One patient out of the seven patients with a non-perforated appendix developed a CSF infection and had their shunt externalized. Three patients out of the four patients that had a perforated appendix had their shunts externalized. Two patients developed pseudocysts. One patient died. According to their findings, there seems to be a slightly higher risk of VP shunt infection following a ruptured appendix [14].

Pumberger and colleagues presented a series of six children, three with a perforated appendix (50%), and three with a non-perforated appendix (50%). None underwent shunt externalization, and none of the patients showed shunt complications related to the surgery or appendicitis. The authors concluded that the VP shunt system may be left in place in the case of appendicitis due to the low risk of infection [9].

Barina et al. looked at five adults, four with perforated appendicitis and one with gangrenous appendicitis. One patient’s VP shunt was discontinued following discovery of a Gram-positive cocci in the peritoneal fluid. No shunt infections occurred, and the authors concluded that the presence of a VP shunt does not increase risk of postoperative complication in patients undergoing appendectomy for appendicitis. The authors did recommend that several precautions be taken to reduce risk of shunt infection, such as taking care to eliminate any concurrent infection prior to elective surgery or administering perioperative antibiotic prophylaxis [4].

Dalfino and colleagues describe a series of seven patients with acute peritonitis, two of whom had a ruptured appendix. During the follow-up interval of 18.5 months no patients developed a VP shunt infection. However, all patients were treated with antibiotics. The authors suggest that externalization of the shunt should be considered only if it presents less risk to the patient than leaving it in place [11].

Hadani and colleagues followed two patients, one with a perforated appendix and one with a phlegmonous appendix. Both patients’ shunts were externalized, and the authors assert that shunt externalization in patients who exhibit acute abdominal symptoms is essential and may prevent unnecessary laparotomy. One of the patients died after developing septicemia. The CSF culture showed growth of *Escherichia coli *[15].

Although the literature shows differing rates of infection and shunt externalization practices, our results demonstrate significant rates of shunt infection with acute appendicitis. Although shunt externalization is not without its risks, externalizing shunts may be able to prevent serious infection, and might be the safest option for these patients [16].

Our analysis was not without limitations. The major limitation of this study was that it was retrospective. Furthermore, due to the nature of the database, we were unable to collect patient-level data on specific outcomes. We were unable to report on radiology information. We do not have information on the type of diagnostic test used for confirmation of disease. The data collected was for billing purposes, not for clinical use, and thus much clinical information is missing. In addition, some misidentification is inevitable in database studies.

## Conclusions

There is a limitation of not being able to determine if the appendix was ruptured or unruptured in this study. Nevertheless, our results suggests that shunt externalization should be strongly considered in patients with appendicitis given high rates of shunt infection. Because shunt externalization is not without its risks, this decision should be made within the clinical context of each individual patient.
